# Mycotoxin Contamination Management Tools and Efficient Strategies in Feed Industry

**DOI:** 10.3390/toxins12080480

**Published:** 2020-07-29

**Authors:** Federica Cheli

**Affiliations:** 1 Department of Health, Animal Science and Food Safety, Università degli Studi di Milano, 20134 Milan, Italy; federica.cheli@unimi.it; 2 CRC I-WE (Coordinating Research Centre: Innovation for Well-Being and Environment), Università degli Studi di Milano, 20134 Milan, Italy

Mycotoxins represent a risk to the feed supply chain with an impact on animal health, feed industry, economy, and international trade. A high percentage of feed samples have been reported to be contaminated with more than one mycotoxin. Multi-mycotoxin contamination is a topic of great concern, as co-contaminated samples might still exert adverse effects on animals due to additive/synergistic interactions of the mycotoxins. Since mycotoxin contamination cannot be completely prevented pre- or post-harvest, precise knowledge of mycotoxin occurrence, repartitioning during technological processes and decontamination strategies are critical and may provide a sound technical basis for feed managers to conform to legislation requirements and reduce the risk of severe adverse health, market and trade repercussions.

Castaldo et al. [1] developed and validated a quantitative method, using an acetonitrile-based extraction and an ultra-high-performance liquid chromatography coupled to high-resolution mass spectrometry (UHPLC-Q-Orbitrap HRMS), for a multi-mycotoxin screening of 28 mycotoxins and identification of other 45 fungal and bacterial metabolites in dry pet food samples. Results showed mycotoxin contamination in 99% of pet food samples and all positive samples showed co-occurrence of mycotoxins with the simultaneous presence of up to 16 analytes per sample.

Strategies must be developed for mycotoxin reduction in feedstuffs. Čolović et al. [2] reviewed the most recent findings on different processes and strategies for the reduction of toxicity of mycotoxins in animals giving detailed information about the decontamination approaches to mitigate mycotoxin contamination of feedstuffs and compound feed, which could be implemented in practice. Authors conclude that there is increasing business interest in the use of feed additives to avoid mycotoxin absorption and the toxic impacts on farm animals. The efficacy of the additives for the distinct mycotoxins and livestock is a critical point and must be proved. It is recommended that cell lines or in vitro models be used in the simulation instead of living experimental animals. In this scenario, a group of papers deals with in vitro models for assessing mycotoxin toxicity and risk mitigation strategies. Xu et al. [3] reviewed different in vitro intestinal epithelial cells (IECs) or co-culture models that can be used for assessing mycotoxin exposure, toxicity, and risk mitigation. Since ingestion is the most common route of mycotoxin exposure, the intestinal epithelial barrier, comprised of IECs and immune cells such as macrophages, represents ground zero where mycotoxins are absorbed, biotransformed, and elicit toxicity. Several articles investigated the efficacy of feed additives as multi-mycotoxin adsorbent by using in vitro gastro-intestinal models. Adunphatcharaphon et al. [4] characterised and analysed acid-treated durian peel (ATDP), an agricultural waste, for simultaneous adsorption of mycotoxins. Results indicated the potential of ATDP as a multi-mycotoxin biosorbent for aflatoxin B1 (AFB1), ochratoxin A (OTA), zearalenone (ZEN), and fumonisin B1 (FB1), but negligible towards deoxynivalenol (DON). Kolawole et al. [5] carried out a study to assess the efficacy of commercially available feed additives with multi-mycotoxin-binding claims. Their capacity to simultaneously adsorb DON, ZEN, FB1, OTA, AFB1, and T-2 toxin was assessed and compared using an in vitro model designed to simulate the gastrointestinal tract of a monogastric animal. Results showed that only one product (a modified yeast cell wall) effectively adsorbed more than 50% of DON, ZEN, FB1, OTA, T-2 and AFB1. The remaining products were able to moderately bind AFB1 but had less, or in some cases, no effect on ZEN, FB1, OTA and T-2 binding. Rejeb et al. [6] characterized a Tunisian clay, before and after calcination, and investigated the effectiveness of the thermal treatment on the adsorption capacity toward AFG1, AFB2, AFG2, and ZEN using an in vitro gastro-intestinal model. The calcination treatment enhanced mainly the adsorption of aflatoxins. Overall results confirm that mycotoxin binders must undergo rigorous trials under the conditions which best mimic the gastrointestinal environment that they must be active in. Claims on the binding efficiency should only be made when such data has been generated.

A few papers reported results of in vivo studies. Kim et al. [7] evaluated yeast cell wall extract efficacy to reduce multi-mycotoxin (AFs, FUM, and DON) toxicity in pigs and improve performance and gut health in pigs. The yeast cell wall extract effects were more evident in promoting gut health and growth in nursery pigs, which showed higher susceptibility to mycotoxin effects, than in growing pigs. Ogunade et al. [8] applied a targeted metabolomics approach to evaluate the effects of supplementing clay with or without *Saccharomyces cerevisiae* fermentation product on the metabolic status of dairy cows challenged with AFB1. Blood was analysed for metabolomic analysis. The study confirmed the protective effects of sequestering agents in dairy cows challenged with AFB1. Moreover, the combination of arginine, alanine, methylhistidine, and citrulline were found to be excellent potential biomarkers of aflatoxin ingestion in dairy cows fed no sequestering agents. The evaluation of mycotoxin biomarker could be an interesting tool for assessing animal exposure to mycotoxin in feed. Gambacorta et al. [9] measured the urinary mycotoxin and mycotoxin biomarker concentrations to assess pig exposure to mycotoxins in Sweden. They found regional differences that were in good agreement with the occurrence of *Fusarium graminearum* mycotoxins in cereal grains harvested in Sweden. From a safety and risk management perspective, the back-calculated levels of mycotoxins in feeds were low with the exception of a few samples that were higher than the European limits.

Paiva Rodrigues at al. [10] carried out an in vitro study to contribute to the knowledge to develop effective anti-mycotoxigenic natural products for reduction of mycotoxigenic fungi and mycotoxins in foods. Authors evaluated the effects of different concentrations of neem oil on the percentage of growth inhibition of six *Aspergillus carbonarius* strains and OTA production. Results indicated that neem essential oil can be considered as an auxiliary method for the reduction of mycelial growth and OTA production.

One paper deals with an important topic: insects as suitable alternative feed for livestock production. Insects have the ability to grow on a different spectrum of substrates, which could be naturally contaminated by mycotoxins. Studies on insect safety as feed ingredients are mandatory for the feed industry. Leni et al. [11] evaluated the mycotoxin uptake and/or excretion in two different insect species, *Alphitobius diaperinus* (Lesser Mealworm, LM) and *Hermetia illucens* (Black Soldier Fly, BSF), grown on naturally contaminated wheat and/or corn substrates (DON, FB1, FB2, and ZEN). No mycotoxins were detected in BSF larvae, while quantifiable amount of DON and FB1 was found in LM larvae. Mass balance calculations indicated that BSF and LM metabolized mycotoxins in forms not yet known, accumulating them in their body or excreting in the faeces. Results indicate that further studies are required in this direction due to the future employment of insects as feedstuff.
